# Integrative modeling of FOXO-mediated autophagy in NSCLC: linking cGAS–STING signaling to IL-6 dynamics

**DOI:** 10.3389/fonc.2025.1689137

**Published:** 2025-11-25

**Authors:** Divya Rani, Shweta Khandibharad, Shailza Singh

**Affiliations:** 1 Systems Medicine Laboratory, Biotechnology Innovation and Research Council (BRIC)-National Centre for Cell Science, Pune, India; 2 Regional Centre for Biotechnology, Faridabad, Haryana, India

**Keywords:** FOXO1, FOXO3A, STING, autophagy, mathematical model, network biology

## Abstract

Lung cancer, particularly non-small cell lung cancer (NSCLC), remains the leading cause of cancer-related mortality worldwide, accounting for approximately 85% of lung cancer cases. Despite therapeutic advancements, the prognosis for advanced-stage NSCLC remains poor due to late diagnosis and high rates of therapeutic resistance. Recent studies have implicated the cyclic GMP-AMP synthase (cGAS)–stimulator of interferon genes (STING) pathway in NSCLC progression, revealing its dual role in innate immune activation and autophagy induction. Concurrently, cGAS–STING activation triggers noncanonical autophagy. We proposed a systems biology framework integrating mathematical model and network biology to elucidate how forkhead box class O (FOXOs) FOXO1 and FOXO3a serve as critical regulators linking cGAS–STING signaling with interleukin-6 (IL-6) in promoting autophagy in NSCLC. Furthermore, sequence, phylogeny, structure, domain, and protein–protein interaction studies identified crucial amino acids and their functions in regulating cGAS–STING– FOXO1 and cGAS–STING–FOXO3a interactions. Our integrative model highlights the complex interplay between immune signaling, metabolic reprogramming, and autophagic regulation in NSCLC. Further findings offer mechanistic insights into the dual role of FOXO proteins in autophagy mediated cancer progression and present potential components for the development of personalized therapeutic strategies aimed at targeting the cGAS–STING–FOXO–autophagy axis.

## Graphical Abstract

Graphical abstract of the cGAS–STING/IL-6 pathway promoting autophagy through FOXOs in NSCLC

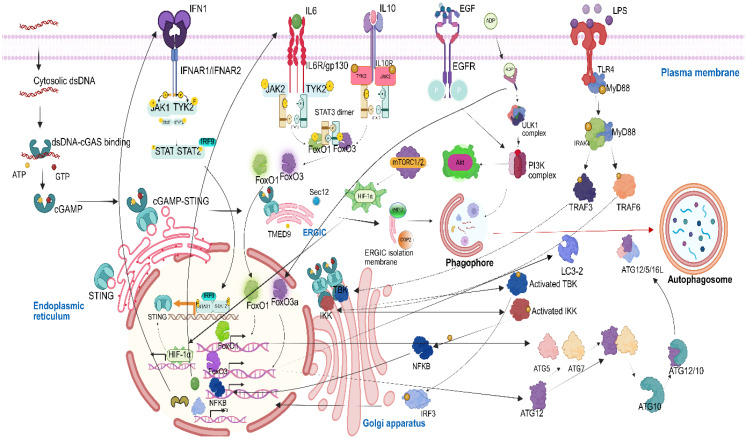

## 1 Introduction

Cancer remains one of the most significant global health challenges of the current time, encompassing more than 100 distinct types, defined by their tissue of origin and molecular characteristics. Over the past few decades, significant progress has been made in understanding the molecular mechanisms of carcinogenesis. Many cancer types still continue to have poor prognosis, especially when diagnosed at advanced stages when the tumor has metastasized to nearby organs as well. Among these, lung cancer has been a major concern due to its high incidence in 2022 with age-standardized incidence rate (ASIR) of 32.1 per 100,000 inhabitants and disproportionately high mortality rate inferred with age-standardized mortality rate (ASMR) of 16.8 per 100,000 (1, 2).

Lung cancer is categorized into two main types: small cell lung cancer (SCLC) and non-small cell lung cancer (NSCLC). NSCLC comprises several histological subtypes, including adenocarcinoma, squamous cell carcinoma, and large cell carcinoma, each with distinct pathological and molecular features and accounts for approximately 85% of all lung cancer cases. Despite advances in early detection and targeted therapies, NSCLC remains a major contributor to global cancer mortality due to its asymptomatic progression, late-stage diagnosis, and high rate of therapeutic resistance. The latest Global Cancer Observatory (GLOBOCAN) data provided by the International Agency for Research on Cancer for the year 2022 estimated that there were approximately 2.48 million new lung cancer cases worldwide, with NSCLC comprising the majority of these diagnosis. In that period, lung cancer was responsible for approximately 1.8 million deaths, representing 18% of all cancer-related fatalities, making lung cancer the leading cause of cancer mortality across the globe (2). Moreover, according to American Cancer Society, the 5-year survival rate of NSCLC patients is approximately 28%, highlighting the urgent need for improved early detection methods and more targeted, personalized treatment approaches (https://seer.cancer.gov/statfacts/html/lungb.html).

Considering the urgency, recent advancements in treatment approaches integrated with chemotherapy, radiotherapy, surgery, and targeted therapy are in application, aiming to bring better outcomes in terms of NSCLC treatment. In the context of metabolic pathways contributing to the development of NSCLC, multiple factors particularly aberrant signaling driven by somatic mutations in the epidermal growth factor receptor (EGFR)-mediated rat sarcoma/rapidly accelerated fibrosarcoma/mitogen-activated protein kinase (RAS/RAF/MAPK), phosphoinositide 3-kinase/protein kinase B (PI3K/AKT), and Janus kinase/signal transducer and activator of transcription (JAK/STAT) pathways have been identified as critical factors leading to tumor survival. Recent studies have also highlighted the emerging role of the cyclic GMP-AMP synthase (cGAS)/stimulator of interferon genes (STING) pathway in NSCLC pathogenesis. Belonging to the nucleotidyltransferase (NTase) superfamily, cGAS, is a cytoplasmic DNA sensor consisting 522 amino acids and has a molecular weight of approximately 60 kDa. The N-terminal of cGAS is positively charged, thus enhancing its affinity toward DNA (3) as DNA, due to the presence of the phosphate group, is negatively charged. The cGAS protein contains sites for modifications, such as phosphorylation and ubiquitination, which regulate the enzyme’s activity as well as stability in the cytoplasm (4). The C-terminal catalytic domain, encompassing the NTase core with male abnormal 21-like protein domain (Mab21) domains, is crucial for 2′,3′-cyclic GMP-AMP (cGAMP) production, DNA binding, and the overall regulation of cGAS function (5).

In 2013, cGAS was recognized as a key cytosolic DNA sensor which detects dsDNA not based on DNA sequence but the length of the nucleotide in various types of cells (3). cGAS is activated in a DNA length-dependent manner, requiring dsDNA of at least 45 base pairs (bp) (6–8) for efficient recognition and robust cGAMP synthesis. DNA fragments shorter than this threshold are significantly less effective at inducing cGAS activation and downstream STING signaling. This length dependency is attributed to the necessity for cGAS to form active dimers or higher-order oligomers upon binding to sufficiently long DNA, which facilitates the conformational changes required for enzymatic activity and immune signaling. Another key adapter protein is STING which is bound to the membrane of endoplasmic reticulum (ER) of *Homo sapiens* and gets activated on binding with cyclic dinucleotides (CDNs) produced by cGAS. The human STING protein consists of 379 amino acids and is primarily localized to the ER (9). Structurally, STING is organized into three key domains: an N-terminal transmembrane domain (residues 1–136) that spans the ER membrane via four α-helical segments; a central cyclic dinucleotide-binding domain (CDN binding domain; residues ~153–340), which facilitates homodimerization and binds endogenous and bacterial cyclic dinucleotides such as cGAMP; and a C-terminal tail that encompass TANK-binding kinase 1 (TBK1) phosphorylation site and IRF3 docking site between ~358–366 and ~374–379, which is essential for downstream signaling through the recruitment and activation of TBK1 and interferon regulatory factor 3 (IRF3).

It is now well established in the literature that cGAS–STING is activated in response to the presence of cytosolic double-stranded DNA (10–12). Upon activation, the canonical pathway is initiated, leading to the induction of type I interferons, e.g., interferon alpha (IFN-α) and pro-inflammatory cytokines such as interleukin-6 (IL-6). Notably, recent findings have revealed that cGAS-STING also promotes autophagy by facilitating the formation of isolation membranes from the ER–Golgi intermediate compartment (ERGIC), ultimately leading to phagophore formation in coordination with various cytoplasmic proteins like secretion-associated Ras-related GTPase activating protein 12 (SEC12) and transmembrane emp24 domain-containing protein 9 (TMED9). Since autophagy has been considered as a double-edged sword, triggering cancer progression in advanced stages, the underlying role of cGAS–STING in inducing autophagy should be studied in depth. Autophagy-related genes (ATGs) are transcribed in the nucleus, which results in the formation of autophagosome from phagophore. Thus, coupling these two pathways may shed an insight to identify the crucial regulators which could be targeted to develop cancer therapies. The onset and regulation of autophagy are governed by interconnected signaling pathways involving multiple kinases, phosphatases, and proteins transcribed by various transcription factors.

Among these regulatory factors, the two forkhead box class O (FOXO) transcription factors FOXO1 and FOXO3a have emerged as pivotal modulators of autophagy. Their involvement in cellular stress responses and transcriptional regulation of ATG genes positions them as important links between oncogenic signaling and autophagic control (13, 14). The four mammalian isoforms of FOXO includes FOXO1, FOXO3a, FOXO4, and FOXO6. These FOXO proteins have been shown to be regulated by multiple factors such as DNA damage, nutrient deprivation, cytokines, and hypoxia. These conditions are commonly seen during tumor progression (15). In mammals, these major FOXO proteins function as transcription factors and bind to the promoter region through their forkhead domain often referred to as a “winged-helix” due to its butterfly-like appearance on X-ray crystallography and nuclear magnetic resonance imaging (16). Majorly affected by their subcellular localization, FOXO proteins undergo various posttranslational modifications (PTMs), such as phosphorylation, ubiquitination, acetylation, methylation, glycosylation, and poly (ADPribosyl)ation (PARylation), leading to either inhibition or activation of downstream target genes. FOXO transcription factors are broadly expressed across various tissues, including the ovary, prostate, skeletal muscle, brain, heart, lungs, liver, pancreas, spleen, thymus, and testes. Given their evolutionary conservation, therapeutically targeting these key proteins holds potential for the development of effective agents to overcome NSCLC.

FOXO3a and FOXO1 are two key members of the FOXO transcription factor family due to their central roles in cellular homeostasis, stress responses, and cancer biology especially in NSCLC. With a molecular weight of 71 kDa, FOXO3a is a 673-amino-acid-long protein (17), playing a vital role in regulating a variety of cellular processes such as apoptosis, autophagy, etc., and is conserved across different species. Under conditions of metabolic stress such as ATP deprivation, FOXO3a undergoes nuclear translocation triggered by the inhibition of the PI3K/AKT pathway where it activates a repertoire of autophagy-related genes like ATG12, LC32/B, etc. These transcriptional events promote the initiation and maturation of autophagosomes, contributing to the survival of NSCLC cells under hypoxia, nutrient deprivation, or therapeutic stress.

FOXO1 is 655 amino acids in length which exhibits overlapping yet distinct regulatory functions as that of FOXO3a. It promotes the transcription of key autophagy genes such as ATG5, ATG7, etc., facilitating autophagosome formation and lysosomal fusion. FOXO proteins are known for their role in regulating cancer (13, 18, 19). However, in NSCLC, both FOXO1 and FOXO3a show context-dependent duality in their function in both early and advanced stages and represent a critical node between cGAS, STING, and autophagy which are triggered in the case of NSCLC. Although both STING and FOXO pathways independently orchestrate stress responses, their mechanistic crosstalk in NSCLC remains largely unexplored. In advanced stages of cancer, FOXO proteins are found to play the role of a tumor promoter (20). This dual scenario necessitates fully comprehending the role of FOXO in cancer especially as a promoter which is still raveled. In order to get insights to the intricate network considering these key proteins and delineate how FOXO-mediated autophagy intersects with STING-driven immune signaling, we utilized systems biology approach to investigate the biological pathways represented in the form of a model system, after which integrative analysis was done to decipher the regulation of crucial proteins in NSCLC.

Under normal physiological conditions, DNA in eukaryotic cells is strictly compartmentalized within the nucleus and mitochondria. However, various stress conditions, such as chromosomal instability, mitochondrial damage, necrotic cells, or bacterial invasion, can disrupt cellular homeostasis, leading to the accumulation of dsDNA in the cytosol. Among these, chromosomal instability, in the case of cancer, emerges as a hallmark of cancer, often associated with tumor progression and metastasis (21). Moreover, as a crucial part of innate immunity, the cGAS–STING pathway is activated. cGAS, being a pattern recognition receptor, detects and binds to cytosolic dsDNA in a sequence-independent but length-depend​​ent manner (3, 22, 23). As soon as dsDNA binds to cGAS, the conformational change in cGAS leads to the binding of cGAS to ATP and GTP present in the cytoplasm and catalyzes them into a cyclic dinucleotide (cGAMP), which comprises both 2′−5′ and 3′−5′ phosphodiester linkages (24–26). For efficient cGAMP synthesis, longer DNA has better potential in activating cGAS, promoting liquid-like droplet formation in which cGAS and dsDNA are concentrated.

cGAMP, the secondary cytoplasmic messenger, is then translocated to the ER and activates STING, after which STING forms tetramers, undergoes high-order oligomerization, and gets translocated to ERGIC. At ERGIC, in the presence of SEC12 and TMED9 protein, some portion of the ERGIC membrane gets separated as an isolation membrane. In the cytoplasm, this ERGIC isolation membrane, in the presence of tryptophan–aspartic acid (WD) repeat domain phosphoinositide interacting protein 2 (WIPI2) and coating protein 2 (COP-2), gets converted into the phagophore. This activation of cGAS–STING leads to a non-canonical autophagy pathway. cGAMP STING, which has been activated then, from ERGIC gets translocated to the Golgi apparatus, where it activates TBK1 and inhibitor of κB kinase (IKK). Activated TBK then phosphorylates IRF3 and leads to its translocation into the nucleus. Activated IKK binds to nuclear factor kappa B (NF-κB) and gets translocated as p65p50 complex. In the inactivated form, phosphorylated IRF3 transcribes interferon alpha (IFN1) and subsequently translocates into the plasma membrane. Furthermore, NF-κB leads to the transcription of IL-6, which also gets translocated to the plasma membrane via the cytoplasm. This leads to an increased expression of IL-6 and IFN1 in NSCLC cells. These cytokines have an inflammatory role in NSCLC, thus promoting poor prognosis and tumor cell survival.

IFN1 binds to interferon alpha/beta receptor subunit 1 and 2 (IFNAR1/2), leading to the activation of IFN1. The activated IFN1 further binds to Janus kinase 1/tyrosine kinase 2 (JAK1/TYK2). As a result of cancer cell immuno-modulation, several researchers have suggested the increased production of interleukin-10 (IL-10) in NSCLC cells (27). Later, IL-10 binds to interleukin-10 receptors (IL-10R1/R2), after which IL-10 signaling gets activated; thus, the JAK1/TYK2 pathway is triggered via two interleukins, i.e., IL-6 and IL-10. Subsequently, the signal transducer and activator of transcription 1 and 2 (STAT1/2) and interferon regulatory factor 9 (IRF9) is activated via JAK/STAT signaling and forms a STAT1/2/IRF9 complex, which is translocated into the nucleus. The STAT1/2/IRF9 complex transcribes the STING gene, which further gets translocated into the ER membrane, thus making a positive feedback loop. The IL-6 induction in the NSCLC also leads to the activation of the JAK2/TYK2 pathway. The activated JAK2/TYK2 further leads to dimerization and activation of STAT3. This STAT3 dimer further dephosphorylates FOXO1 and FOXO3a present in the cytoplasm, leading to their translocation into the nucleus. NSCLC tumor cells rely on glycolysis as their metabolic process, leading to the rapid production of adenosine diphosphate (ADP), the phenomenon commonly referred to as the Warburg effect. When ADP is increased in the nearby cells, it enters the NSCLC cells and directly binds to adenosine monophosphate (AMP) gamma and further leads to the activation of Unc-51 like autophagy activating kinase 1 (ULK1) complex, a major protein involved in autophagy. This ADP/AMP gamma complex in activated form also regulates FOXO3a in the nucleus (28). The activated ULK1 complex then activates PI3K, another key protein involved in the formation of phagophore. Epidermal growth factors (EGF) are present in more than 50% of NSCLC patients and play a primary role in poor prognosis (29). EGF, upon binding with EGFR, gets activated and phosphorylates PI3K, thus again promoting the activity of PI3K to lead to phagophore formation. Apart from phagophore formation, PI3K further activates AKT. Upon phosphorylation, AKT activates the mechanistic target of rapamycin complex 1 (mTORC1). mTORC1 then activates a key transcription factor involved in cellular response to low oxygen levels, hypoxia-inducible factor 1-alpha (HIF-1α). Translocation of HIF-1α into the nucleus leads to the transcription of the STING protein. Lipopolysaccharide (LPS) binds to TLR4, which then activates myeloid differentiation primary response gene 88 (MYD88). MYD88, on activation, phosphorylates interleukin-1 receptor-associated kinase 4 (IRAK4), a serine/threonine kinase. IRAK4 then phosphorylates TNF receptor-associated factor 3 (TRAF3) and TNF receptor-associated factor 6 (TRAF6). TRAF3, then, further recruits TBK1 to the Golgi apparatus, whereas TRAF6 recruits IKK to the Golgi apparatus, the two key proteins, activated by cGAMP-STING for IL-6 and IFN1 transcription.

At the advanced NSCLC, emerging evidence from multiple systems indicates that FOXOs orchestrate the expression of genes involved in autophagy, which, in turn, fuel cancer cells to survive in nutrient-deprived conditions and combat the stress caused during chemotherapy. The activation or deactivation of FOXO proteins depends on their shuttling between the cytoplasm and the nucleus. The mathematical model depicts that when the FOXO1 and FOXO3a proteins are triggered in NSCLC, FOXO3a leads to the transcription of microtubule-associated protein 1 light chain 3 beta (LC3-2) and ATG12. FOXO1 leads to transcription of the ATG5 and ATG7 genes. ATG7 binds to ATG12, and the complex then activates ATG10, leading to the formation of ATG12/10. This ATG12/10 binds to ATG5, and an ATG12/5 complex is formed, which further binds to ATG16L. The ATG12–ATG5–ATG16L1 complex acts as an E3-like ligase in the autophagy pathway, crucial for nucleating and expanding the phagophore (the initial isolation membrane) into a mature autophagosome by scaffolding the lipidation process. The lipidated LC3-2B then leads to membrane curvature and expansion and further sealing of the phagophore and its closure into an autophagosome.

The study aims to unite FOXO-regulated autophagy and STING-mediated immune signaling within a single dynamic framework in NSCLC. By bridging molecular stress pathways with immune-modulatory outputs, the model provides novel mechanistic insights into how tumor cells adapt to stress and how these adaptations might be exploited therapeutically for NSCLC treatment.

## 2 Methodology

### 2.1 Data collection

To explore the regulatory role of FOXO1 and FOXO3a in signaling networks involved in cGAS–STING-mediated autophagy, information was gathered from the KEGG pathway and from relevant scientific literatures in PubMED (30). The integration of literature-derived and publicly available datasets enabled the development of a comprehensive network describing FOXO–STING–autophagy signaling dynamics in NSCLC (31) and drawing the connectivity among various pathways surrounding cGAS–STING-driven autophagy, bridging existing gaps in understanding how these complex pathways facilitate intricate tumorigenesis in NSCLC.

### 2.2 Reconstruction of mathematical model for FOXO3a-mediated cGAS–STING autophagy

To elucidate the regulatory role of FOXO1 and FOXO3a in immune signaling and autophagy, with the cGAS–STING signaling axis in NSCLC, a mathematical model was reconstructed. Using SimBiology toolbox from MATLAB v7.11.1.866, the model was built comprising different compartments depicting cell organelles, including plasma membrane (PM), cytoplasm, nucleus (N), ER, Golgi apparatus, and ERGIC containing different proteins which are involved in FOXO-mediated autophagy.

SimBiology is a programmatic tool utilizing systems biology mark-up language (SBML) format to determine and analyze mechanisms underlying the complex and dynamic behavior of the species of biological systems and how they interact under defined parameters. The protocol which was used to construct, simulate, and analyze the mathematical model was taken from Khandibharad et al. (32), and the goal of quantitative modeling approach was to gain mechanistic insights into the critical proteins involved in FOXO-mediated autophagy involving cGAS–STING and decipher their interactions when triggered by external signals.

In the section of block diagram editor, different entities like compartment, species, and reactions are present. Compartments depict cell organelles; species were used to depict reactants and products, after which rate law was defined between them. The reactions are assigned based on specific rate laws. For association/dissociation/translocation, we used Law of Mass action; for enzyme kinetic, Michaelis–Menten equation was used; and for reactions involving gene expression, we used Hill kinetic equation. Considering these parameters, values are provided with respective units, and the initial concentration was considered between 10^3^ and 10^6^ signaling molecules (33). The reconstructed mathematical model discretely mimicked the parameters of cGAS–STING signaling model system in NSCLC.

### 2.3 Mathematical model analysis

The mathematical model is analyzed using different system-based approaches like sensitivity analysis, principal component analysis (PCA), flux analysis, and model reduction. These techniques help identify the most important components and reactions that control how the system behaves and what are the most crucial reactions that have a major impact on the overall behavior of the model.

#### 2.3.1 Simulation

In simulation, the SimBiology tool box utilizes a stiff ordinary differential equations (ODE15s) based on variable-order numerical differentiation formulas (NDFs) to stimulate the reconstructed mathematical model. Keeping the absolute and relative tolerance as default, the behavior of each component was obtained in the form of state vs. time graph further defined along with concentration (in molecules) and parameter within the stimulated time of 100 s.

#### 2.3.2 Sensitivity

Sensitivity analysis is a mathematical approach that enhances the understanding of mathematical model’s robustness by quantifying how variations in external parameters influence model behavior, thereby identifying the key components that predominantly govern the model’s outcomes. This means that the reconstructed mathematical model is able to maintain its accuracy and reliability even when the input data, assumptions, or parameters are altered by building the confidence in the model.

Local and global sensitivity analysis approaches are the two commonly applied sensitivity analysis in systems biology (34). Local sensitivity analysis gives insights on how small changes in the input parameters such as reactant concentration, reaction rate, etc., on a mathematical model influence output behavior such as product concentration. On the other hand, global sensitivity analysis approaches are applied to understand how the model outputs are affected by large variations of the model input parameters.

We have performed local sensitivity to understand the impact of a single component and its associated parameters on the overall model dynamics by keeping each component as input parameter one by one and others as output. It is represented at the time-dependent derivatives as Eq. dx/dy, dx/dz, where the components in the model are considered as “x” and other parameters are considered as “y” and “z”. The sensitivity outputs and sensitivity inputs are mentioned as numerator and denominator to calculate sensitivity using SUite of Nonlinear and DIfferential/ALgebraic equation Solvers (SUNDIALS). It integrates the original ordinary differential equations (ODE) and computes the time-dependent sensitivities for each species state with respect to each parameter value.

#### 2.3.3 Principal component analysis

Principal component analysis (PCA) is used to simplify the large set of values characterized by numerous inter-correlated quantitative dependent variables and helps highlight which components in the system have the most influence in the overall mathematical model by giving a principal component score to each component to reduce the complexity of the data and removes background noise, thus making our data more robust and reproducible. A high PCA score means that a component is very sensitive and if it is targeted, the system could collapse.

We calculated PCA in MATLAB using the function: score_coefficient = princomp(A).

Here A is an m × n matrix containing sensitivity scores of each component with respect to local sensitivities in the system.

#### 2.3.4 Flux analysis

This constraint-based study is based on the principle of mass conservation in the biological network which utilizes the stoichiometric matrix (35) to determine how much each reaction is contributing in the flow of the entire mathematical model and how the flow of these components is balanced at steady state, which helps us understand the productivity of each reaction and overall system behavior or phenotype. We have adopted comparative flux analysis to determine the productivity of each reaction in mathematical model. The value of flux for each reaction is obtained in (mol/s) using COmplex PAthway Simulator (COPASI) v4.11, a modeling stimulator which defines the flux of every reaction present in our reconstructed mathematical model. Based on high flux rates ranging between 215,000 and 500 mol/s, the top 27 reactions are mechanistically sorted to understand the highly contributing reactions.

#### 2.3.5 Model reduction

Defined as a computational biology approach, model reduction aims to reduce the computational complexity of reconstructed mathematical models so that the behavior of significant kinetic equations can be studied among the complex reactions by eliminating transient and intermediate reactions that do not contribute significantly to network output (36).

For model reduction, three parameters which we had calculated previously have been combined, the reactions with the value of sensitivity close to one in sensitivity analysis (to see which reactions affect the system the most), highest flux values in flux analysis (to sort out the reactions with the highest rate of flow), and concentration through time series (to find key components and reactions). The data was used as an input in Sigmaplot (15.0) to generate a 3D mesh graph representing quasi-potential landscape. The purpose of model reduction is to eliminate spurious parameters and reactions which had least contribution in maintaining a stable steady state of the biological network.

#### 2.3.6 Crosstalk identification

In the reconstructed mathematical model, crosstalk point refers to the interaction between biological networks connected via particular signaling components, which influences the model dynamics and acts as a node in regulating signaling. With the help of crosstalk analysis, the relative importance of each component in the network can be quantitatively assessed in the context of NSCLC. The crosstalk score quantifies the extent to which the given component participates in the network and could serve as potential candidates for therapeutic intervention.

The total degree of an individual node – degree of a node within its pathway = non-zero value of the crosstalk point.

#### 2.3.7 Network analysis of the reconstructed mathematical model

In order to analyze the complex sets of interactions between different components in our reconstructed mathematical model, biological entities were represented as nodes, and the functional entities which connects them were referred to as edges (37). This can be well understood by Graph theory postulated by Leonhard Euler, where the networks, represented in terms of graph, can be used to capture interactions between the molecules (38). For the connectivity analysis of the network, Cytoscape (3.10.3) along with its plugins BiNGO and CytoHubba have been utilized. CytoHubba allows an analysis of the biological network using different parameters such as closeness, betweenness, bottleneck, degree, clustering coefficient, maximum neighborhood component (MNC), density of maximum neighborhood component (DMNC), eccentricity, edge percolated component (EPC), maximal clique centrality (MCC), radiality, and stress to elucidate the network properties. In the reconstructed mathematical model, we visualized overrepresented categories after correction, and for this, Biological Networks Gene Ontology tool (BiNGO), a plugin for Cytoscape, has been used. For analysis, a hypergeometric test was selected as the statistical test, and Benjamin and Hochberg false discovery rate (FDR) was applied for correction. Using whole annotation as a reference set and GO_Biological_Process as an ontology file, BiNGO assesses the overrepresentation of gene ontology (GO) categories and describes gene products in terms of biological processes, molecular functions, and cellular components. After selecting *Homo sapiens* as organism/annotation, the functional roles of genes during network analysis was determined (39).

#### 2.3.8 Structure prediction of cGAS, STING, FOXO1, and FOXO3a with domain analysis and molecular docking

Considering that cGAS–STING-mediated FOXO3a-induced autophagy is responsible for the triggered growth and persistent survival of tumor cells in NSCLC, it is necessary to elucidate the structure of target proteins to better understand the function and three-dimensional conformation, active domains, and interaction interfaces will provide critical insights into their functional mechanisms and regulatory dynamics.

In this study, we have performed homology modeling approach for the prediction of 3D structure of both cGAS and STING from *Homo sapiens*. The query templates of cGAS (accession ID: Q8N884) and STING (accession ID: Q86WV6) were downloaded from NCBI website for *Homo sapiens*. The templates identified for cGAS from protein data bank (PDB) was PDB ID-4Km5 and for STING was PDB ID-8GT6; using these templates and Modeller 9.18, a structural model via homology modeling was performed (40). The parameters for selecting the best models were done by the assessment of the structures by calculating the root mean square distance (RMSD) via PyMOL 1.7.4.4, derivation of Ramachandran plot via PDBSum generate and calculation of z-score via ProSA-web. For proteins FOXO1 (accession ID: Q12778) and FOXO3a (accession ID: O43524) for *Homo sapiens*, *ab initio* modeling using Robetta server was utilized (available online: https://robetta.bakerlab.org/) and validated based on the z-score using ProSA-web (available online: https://prosa.services.came.sbg.ac.at/prosa.php) and Ramachandran plot using PDBSum generate (available online: https://www.ebi.ac.uk/thornton-srv/databases/pdbsum/Generate.html). The domains of each protein was mapped based on the literature available (41–43). To understand the functions of protein, it is necessary to get an insight on how the proteins interact; so, in order to check the important residues of cGAS–STING with its interacting partners, protein–protein interaction was employed. For the exploration of protein–protein interactions between cGAS and STING and further with cGAS–STING and FOXO3a and FOXO1 as well, the ClusPro2.0 web server (available online: https://cluspro.org/) was used.

#### 2.3.9 Phylogenetic analysis

The protein sequences of FOXO1, FOXO3a, FOXO4, and FOXO6 for *Homo sapiens* and *Mus musculus* were downloaded from the National Centre for Biotechnology Information (NCBI, United States) in.fasta format. The phylogeny analysis was performed for the entire FOXO family members derived from *Homo sapiens* and *Mus musculus*. Phylogeny was also performed for FOXO3a proteins and FOXO1 proteins of *Homo sapiens* and *Mus musculus* together as well as separately for the two species. Multiple sequence alignment (MSA) is a bioinformatics approach to generate evolutionary tree where the evolutionary closely related sequences are kept together. Using Clustal Omega (available online: https://www.ebi.ac.uk/jdispatcher/msa/clustalo?stype=protein), the.fasta files were converted to.nexus format to create an alignment file for Bayesian inference phylogeny. MrBayes v3.2.6 was utilized for Bayesian phylogenetic analysis, and programming sequences for generations using Metropolis-coupled Markov chain Monte Carlo (MCMC) was done for each nexus file until the average standard deviation of split frequencies reached its lowest. Then, the run with least average standard deviation of split frequency was chosen to visualize the phylogenetic tree as a cladogram using a java-based application, Figtree v1.4.4 (44).

#### 2.3.10 Statistical coupling analysis

To gain a comprehensive understanding of the structural, functional, and evolutionary relationships of key proteins involved in immune and cellular regulation, we performed statistical coupling analysis (SCA) for the amino acid sequences of cGAS, STING, FOXO1, and FOXO3a for two organisms—*Homo sapiens* and *Mus musculus*. The domains in the protein structure consist of amino acid interactions, comprising statistically significant co-evolving residues termed as sectors (45). These sectors are functionally distinct divisions and are represented in blue, green, and red colors, providing insights into the different biochemical properties of the protein and the conservation of functional sites across species (46). The aligned file was converted to the Pearson/FASTA format using Clustal Omega, and SCA was performed using the SCA toolbox in MATLAB R2020a (The MathWorks, Inc., Torrance, CA, USA). The location of amino acid residues within their specified sectors was determined using Dij values, the coupling scores used to quantify the degree of co-evolution between amino acid residues in multiple sequence alignments. The Dij cutoff threshold is often applied between the range of 0.2 and 0.5 to remove noise due to limited sampling of the sequences and retain statistically significant correlations by balancing the combination of sensitivity as well as specificity. To analyze the amino acid residues of proteins FOXO1 and FOXO3a for *Homo sapiens* and *Mus musculus*, the cutoff was set to 0.5. For cGAS, the cutoff was set to 0.5, and for STING protein the cutoff was set to 0.2. SCA was also performed by combining all members of FOXO protein family that are FOXO1, FOXO3a, FOXO4, and FOXO6 for *Homo sapiens* and *Mus musculus* with a cutoff value of 0.2.

By analyzing Dij values, it was attempted to understand the link between the residues and its contribution in protein function. In addition, the conservation of residues with regard to the position was estimated to understand how consistently a particular amino acid is preserved at a given position across all aligned sequences. Highly conserved residues indicate their functional or structural importance, as evolutionary pressure maintains their identity to preserve protein function (47).

#### 2.3.11 Immunofluorescence

Immunofluorescence staining was performed on human NSCLC cell lines H1299, H1975, and A549. H1299 cells and H1975 cells were grown in Roswell Park Memorial Institute medium (RPMI) 1640 media, and A549 cells were cultured in Ham’s F-12 (HF12) media, each supplemented with 10% fetal bovine serum (Gibco) and penicillin–streptomycin (Himedia) (complete growth media) in tissue culture flasks until reaching approximately 90% confluency after incubation in a humidified incubator at a temperature of 37°C at 5% CO_2_ concentration. The cells were then dissociated from the flask using trypsin and seeded into 96-well coverslip bottom plates at a density of 1 × 10^4^ cells per well in 200 μL of complete growth medium. For each cell line, untreated cells were used as control, while cells treated with recombinant human IL-6 protein (Thermo Fisher 200-06) at a final concentration of 0.2 ng/μL for 24 h were used as IL-6-induced sample. After 24 h of IL-6 stimulation, the cells were washed thrice with 200 μL phosphate buffer saline (PBS) and fixed with 4% paraformaldehyde for 20 min at room temperature in the dark and later washed with 200 μL phosphate buffer saline (PBS). Blocking was performed with 3% bovine serum albumin (BSA) in phosphate-buffered saline triton-X (PBST) for 30 min. Subsequently, the cells were incubated for 2 h with primary antibodies against IL-6 (Invitrogen, #701028) and STING (Cell Signaling Technology, #13647). Each primary antibody (1:1,000) was added across the three cell lines in control and IL-6 induction conditions. After incubation, the cells were washed and incubated for 1 h at room temperature in the dark with anti-rabbit secondary antibody (50 μL/well) conjugated with Alexa Fluor 488 and phalloidin. Then, the cells were washed thrice with PBST and stained with 4′,6-diamidino-2-phenylindole (DAPI) at the concentration of 1 μg/μL and incubated for 20 min in the dark. After washing thrice with PBST and twice with deionized water, the samples were acquired using Olympus FV3000, a LSCM with a ×60 oil immersion objective lens. The analysis of protein for each well was done using fluorescent intensity measurement obtained via ImageJ 1.53 where Green channel fluorescence corresponding to the target protein was quantified by outlining individual cells to quantify the expression of IL-6 and STING.

#### 2.3.12 Statistical analysis

The mean fluorescence intensity values (arbitrary units, a.u.) were statistically analyzed using a two-way ANOVA, followed by Sidak’s multiple-comparisons test to adjust for type I error. Data is presented as the mean ± standard deviation (SD), where *p*-value <0.05 was considered statistically significant. Asterisks denoted the statistical significance for differences, with *p*-value less than 0.05 (*), *p*-value less than 0.01 (**), *p*-value less than 0.001 (***), and *p*-value less than 0.0001 (****). The graphs were plotted using GraphPad Prism version 9.0.0 (121).

## 3 Results

### 3.1 Reconstructed mathematical model and simulation

**Figure 1:**
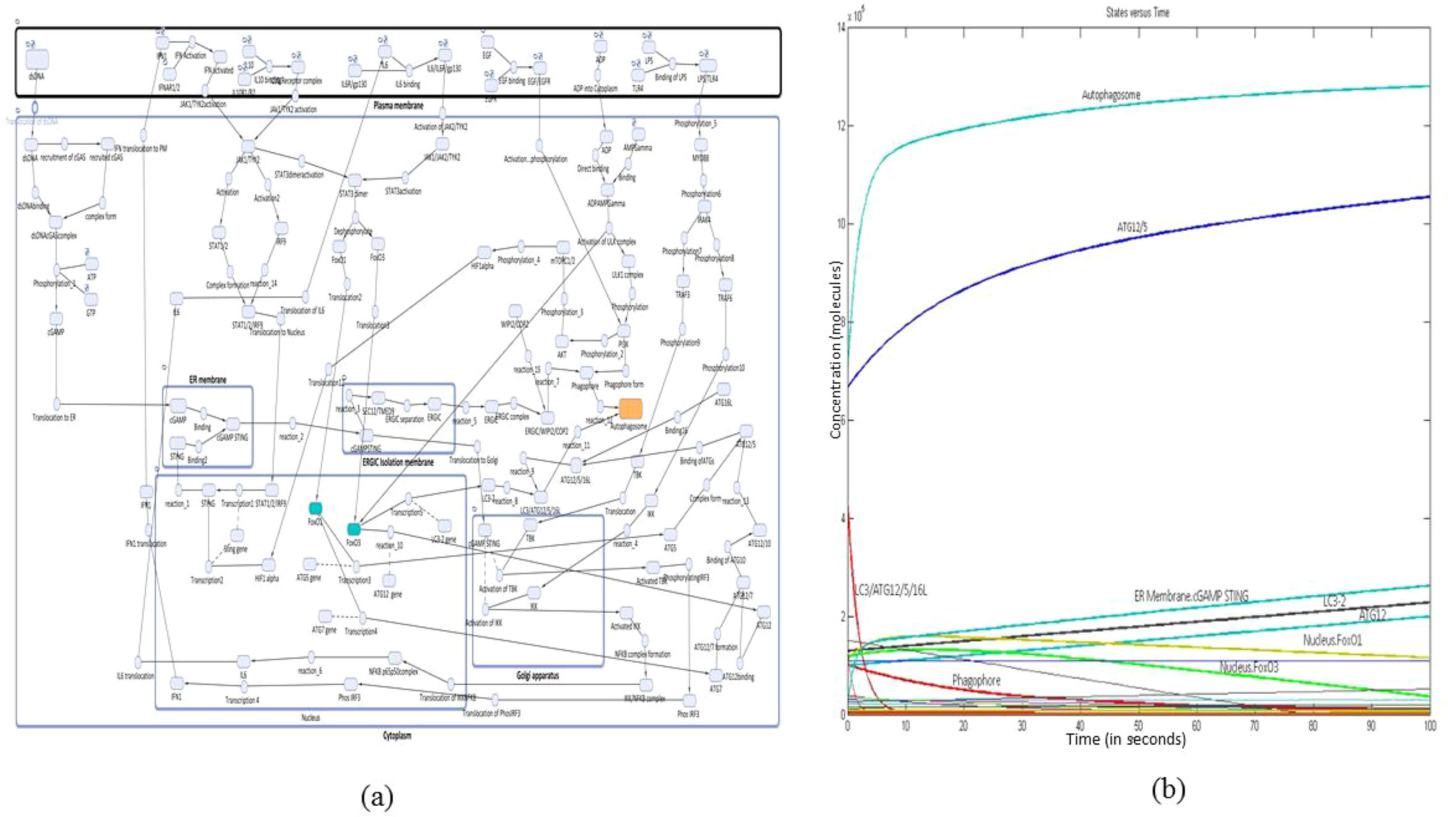
Reconstructed mathematical model and simulation. **(a)** Reconstructed mathematical model of cGAS–STING/IL-6 pathway promoting autophagy through FOXOs in NSCLC. **(b)** Simulation of the reconstructed mathematical model to obtain a concentration-versus-time plot at 100s.

The reconstructed mathematical model of cGAS–STING signaling promoting autophagy in NSCLC was generated with six compartments including cytoplasm, PM, ER, Golgi apparatus, and ERGIC with 72 species and 83 reactions and was simulated for 100 s with a Stiff Deterministic ODE15s solver (SimBiology toolbox). The mathematical model depicts the cascade triggered by the interaction between cGAS present in the cytoplasm and dsDNA which enters the cytoplasm from NSCLC cell, leading to the conformational change in cGAS, with the transition leading to the synthesis of cGAMP. This cGAMP on binding with STING present on the ER membrane leads to the separation of ERGIC as an isolation membrane for phagophore formation. Meanwhile, the activated cGAMP STING also leads to the activation of TBK1 and IKK in the Golgi apparatus, the two proteins which have been recruited via IRAK4-TRAF3 pathway. Activated TBK1 phosphorylates IRF3 present in the cytoplasm which on translocation to the nucleus transcribes IFN1. Subsequently, activated IKK forms a complex with NF-κB complex which in the nucleus transcribes IL-6. This IFN1 along with IL-6 via JAK1/TYK2 pathway activates STAT1/2 dimer and STAT3 dimer. STAT1/2 dimer on activation binds with IRF9, which leads to the transcription of STING. The STAT3 dimer translocates FOXO1 and FOXO3a to the nucleus where they transcribe ATGs, which play a crucial role in the formation of autophagosome from phagophore. The autophagosome is also induced in response to ATP depletion. This energy deficit is attributed to the heightened metabolic demand required to sustain the rapid proliferation characteristic of NSCLC cells. The pathway which is involved is PI3K/AKT/mTORC1 pathway, where PI3K contributes to the initiation of phagophore formation. This PI3K is activated by ligand-bound EGF, and downstream ULK1 complex further activates HIF-1α through mTORC1/2 signaling. Subsequently, HIF-1α upregulates the expression of STING which has an implication in the promotion of phagophore biogenesis. Since the genes transcribed by FOXO1 and FOXO3a are mainly involved in autophagy, the entire mathematical model correlation depicts that there is enhanced autophagy process which is mediated by cGAS–STING complex in the presence of FOXO3a and FOXO1 proteins (**Figure 1a**). The simulation analysis revealed that the major proteins produced at the 100-s simulation include autophagosome, ATG12/5, ER membrane cGAMP STING, LC3-2, ATG12, and FOXO1 and FOXO3a which are present in the nucleus (**Figure 1b**).

### 3.2 Principal component analysis

**Figure 2:**
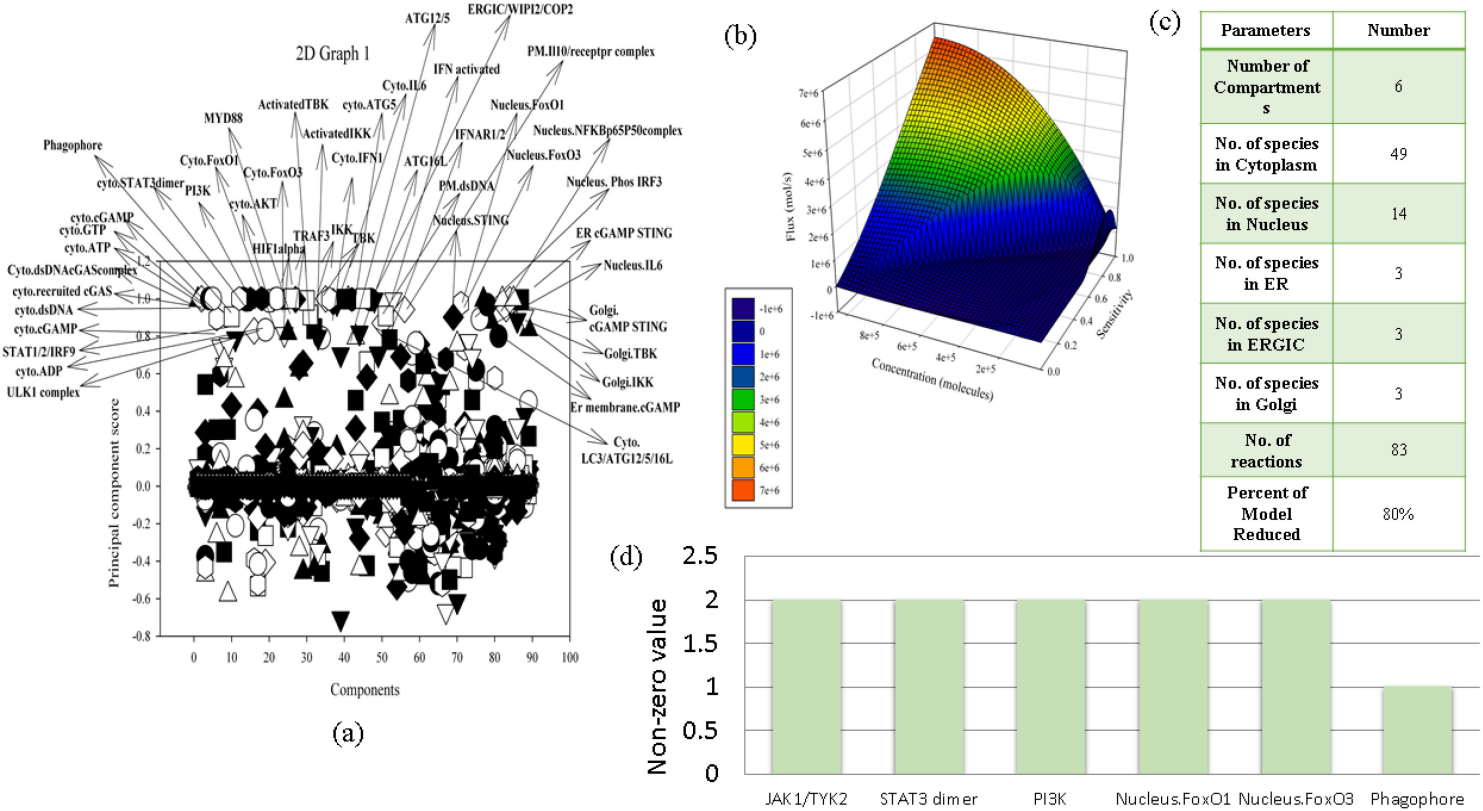
Mathematical model analysis. **(a)** PCA. **(b)** Quasi-potential landscape. **(c)** Summary of reduced model. **(d)** Crosstalk point analysis.

Since our model consists of various signaling proteins, we analyzed local sensitivity analysis to obtain sensitivity coefficients which were further used to perform PCA to churn out the crucial species (**Supplementary File S1**). The key components identified through PCA analysis with PCA score between 0.8 and 1.2 included the following: ULK1 complex, cytoplasmic ADP, STAT1/2/IRF9, cytoplasmic cGAMP, cytoplasmic recruited cGAS, cytoplasmic dsDNA cGAS complex, cytoplasmic ATP, cytoplasmic GTP, phagophore, cytoplasmic STAT3 dimer, PI3K, cytoplasmic FOXO1, cytoplasmic FOXO3a, cytoplasmic AKT, MYD88, HIF1 alpha, TRAF3, IKK, TBK, cytoplasmic IFN1, activated TBK, activated IKK, cytoplasmic IL-6, ATG16L, plasma membrane dsDNA, nucleus STING, ATG12/5, IFN activated, IFNAR1/2, nucleus FOXO1, nucleus FOXO3a, ERGIC/WIPI2/COP2, plasma membrane IL-10 receptor complex, nucleus NFKBp65p50 complex, nucleus phosphorylated IRF3, endoplasmic reticulum cGAMP STING, nucleus IL-6, Golgi cGAMP STING, Golgi TBK, Golgi IKK, endoplasmic reticulum cGAMP, and cytoplasmic LC3/ATG12/5/16L. Through PCA, it was revealed that cGAMP STING, nucleus and cytoplasmic FOXO1 and FOXO3a, nuclear STING, and ATG12/5 are one of the key determinants in the cGAS STING pathway, mediating the autophagy process in the reconstructed NSCLC model (**Figure 2a**).

### 3.3 Flux analysis

To understand the dynamic behavior and directionality of reactions within the mathematical model, flux analysis was performed. Key reactions were identified based on their flux values, with significant fluxes reaching up to 500 mol/s. The reactions having high flux rates include autophagosome formation upon activation of LC3/ATG12/6/16L and phagophore and the translocation of FOXO1 and FOXO3a from the cytoplasm to the nucleus (**Table 1**). The list of reactions along with their flux is shown in **Supplementary File S2**.

**Table 1:** High-flux reactions identified from the reconstructed mathematical model.

Reaction	Flux (mol/s)
LC3/ATG12/5/16L -> Autophagosome	215,000
STING{“ER membrane”} -> “cGAMP STING”{“ER membrane”}	119,785.7388
FoxO1{Cytoplasm} -> FoxO1{Nucleus}	15,962.46
ATG12/10 -> ATG12/5	8,870.4
ATG5 -> ATG12/5	7,884.8
Phagophore -> Autophagosome	5,050
FoxO3{Cytoplasm} -> FoxO3{Nucleus}	4,213.739334
STAT1/2/IRF9{Cytoplasm} -> STAT1/2/IRF9{Nucleus}	3,520.963037
ATG12/7 -> ATG12/10	3,195.52
IFN1{Nucleus} -> IFN1{Cytoplasm}	2,187.30438
IFN activated -> JAK1/TYK2	1,986.823901
IL6{“Plasma membrane”} + IL6R/gp130 -> IL6/IL6R/gp130	1,721.04
STING{Nucleus} -> STING{“ER membrane”}	1,393.470384
cGAMP{Cytoplasm} -> cGAMP{“ER membrane”}	1,327.578
FoxO1{Nucleus} + “ATG5 gene” -> “ATG5 gene” + ATG5	1,114.342414
FoxO3{Nucleus} + “LC3–2 gene” -> LC3-2 + “LC3–2 gene”	995.7870612
ATG12 gene + FoxO3{Nucleus} -> ATG12 + “ATG12 gene”	995.7870612
HIF1alpha -> “HIF1 alpha”	948.9610392
IFN1{Cytoplasm} -> IFN1{“Plasma membrane”}	803.3688
ERGIC/WIPI2/COP2 -> Phagophore	803.0784
ERGIC{“ERGIC Isolation membrane”} -> ERGIC{Cytoplasm}	759.64849
STAT3 dimer -> FoxO1{Cytoplasm} + FoxO3{Cytoplasm}	595.3331605
STAT1/2/IRF9{Nucleus} + “Sting gene” -> STING{Nucleus} + “Sting gene”	587.1056789
HIF1 alpha + “Sting gene” -> STING{Nucleus} + “Sting gene”	557.6449384
IL6{Cytoplasm} -> IL6{“Plasma membrane”}	540.5768064
Phos IRF3{Cytoplasm} -> “Phos IRF3”{Nucleus}	501.8405937
recruited cGAS -> dsDNAcGAScomplex	501

### 3.4 Model reduction

Since our mathematical model is driven by a combination of discrete parameters, a quasi-potential landscape models the dynamics of the system and removes the extraneous species and parameters from the reconstructed mathematical model in order to make it more robust and to predict the model outcomes. The quasi-potential landscape displayed a dome-shaped pattern representing the distribution of high flux, high concentration, and high sensitivity at the top of the dome (**Supplementary File S3**). At the bottom of the graph, the three quantitative parameter values reduce subsequently. Since we have filtered out 12 reactions considering all of the parameters to be the highest among 83 reactions, the model is reduced by 86% (**Figures 2b, c**).

### 3.5 Crosstalk points

In our mathematical model, we came across six crosstalk points which are JAK1/TYK2, STAT3 dimer, PI3K, nucleus FOXO1 and FOXO3a, and phagophore. These components act as connecting bridges between multiple signaling pathways in the model, assisting the model to decide between alternative fates such as survival, apoptosis, inflammation, or autophagy. Hence, in the context of NSCLC, we can determine the activity of proteins which are crosstalk points and target them to understand their behavior in different phenotypes. JAK1/TYK2 was acting as a crosstalk point between IFN1 and IL-10 pathway, STAT3 was acting as a crosstalk point between IL-6 and IL-10 pathway, PI3K was acting as a crosstalk point between EGF and ADP pathway, FOXO1 was acting as a crosstalk point between IL-6, IL-10 and autophagy pathway, FOXO3a was acting as a crosstalk point between EGFR, ADP, IL-10, IL-6, LPS, and autophagy pathway, and phagophore was acting as a crosstalk point between ADP, autophagy, and cGAS–STING pathway (**Figure 2d**).

### 3.6 Network analysis

**Figure 3:**
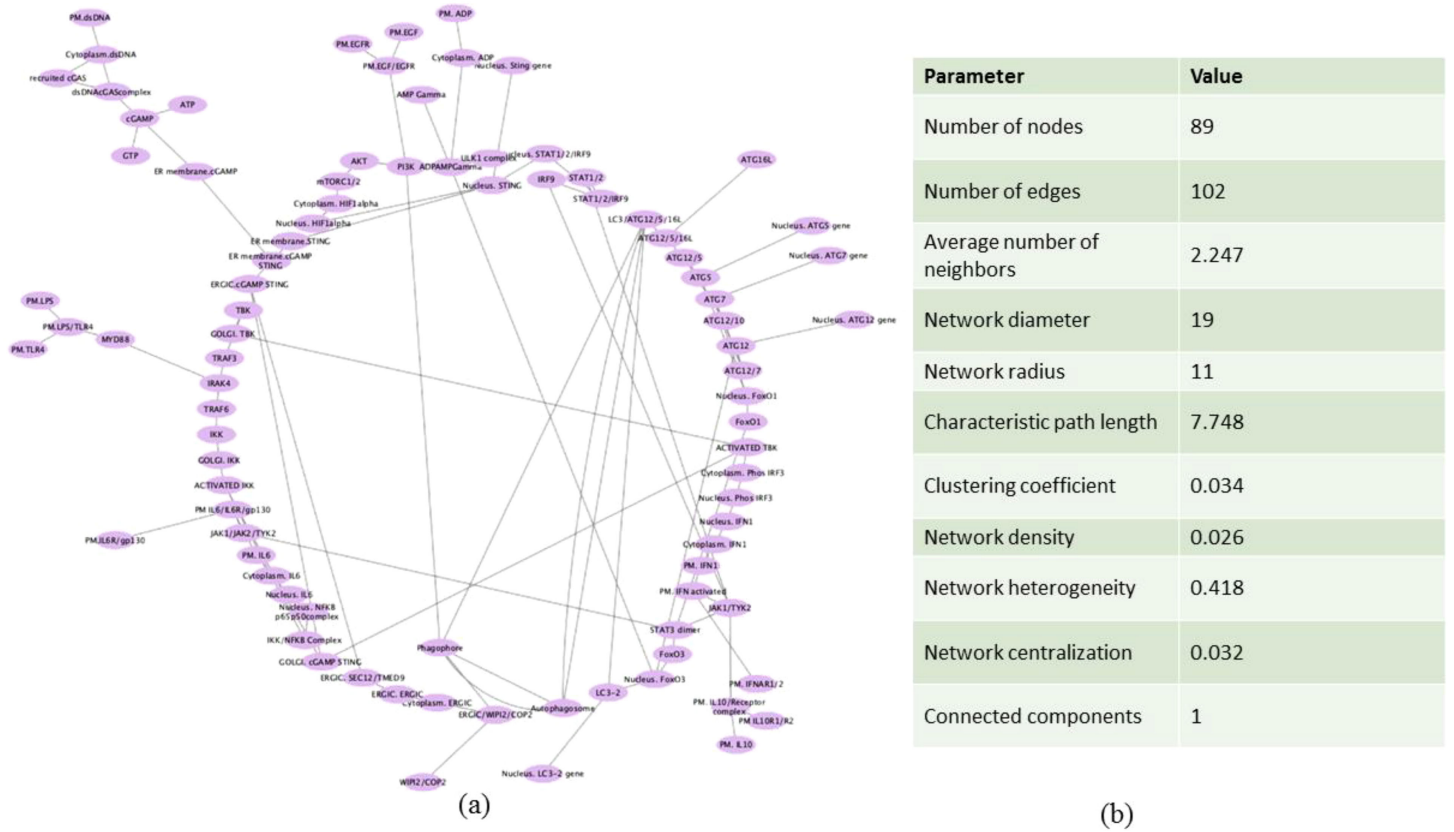
Network construction of the mathematical model. **(a)** Circular layout representation of the mathematical model in the form of network. **(b)** Table showing the parameters of the analyzed network.

In order to view and examine the built model connectivity, we used Cytoscape for network building. The components which were reactants were designated as source and the products were designated as target (**Supplementary File S4**). The network is represented in a circular layout, and the network was subsequently analyzed. The resulting network had an average node degree of 2.247, 89 nodes, and 102 edges. The network radius, or the shortest distance from the most central node, was identified to be 11, while the network diameter, or the longest shortest path between any two nodes, was determined to be 19. The network was found to have a moderate degree of local interconnectivity, as indicated by the clustering coefficient of 0.034, which measures how much nodes tend to cluster together (**Figures 3a, b**).

**Figure 4:**
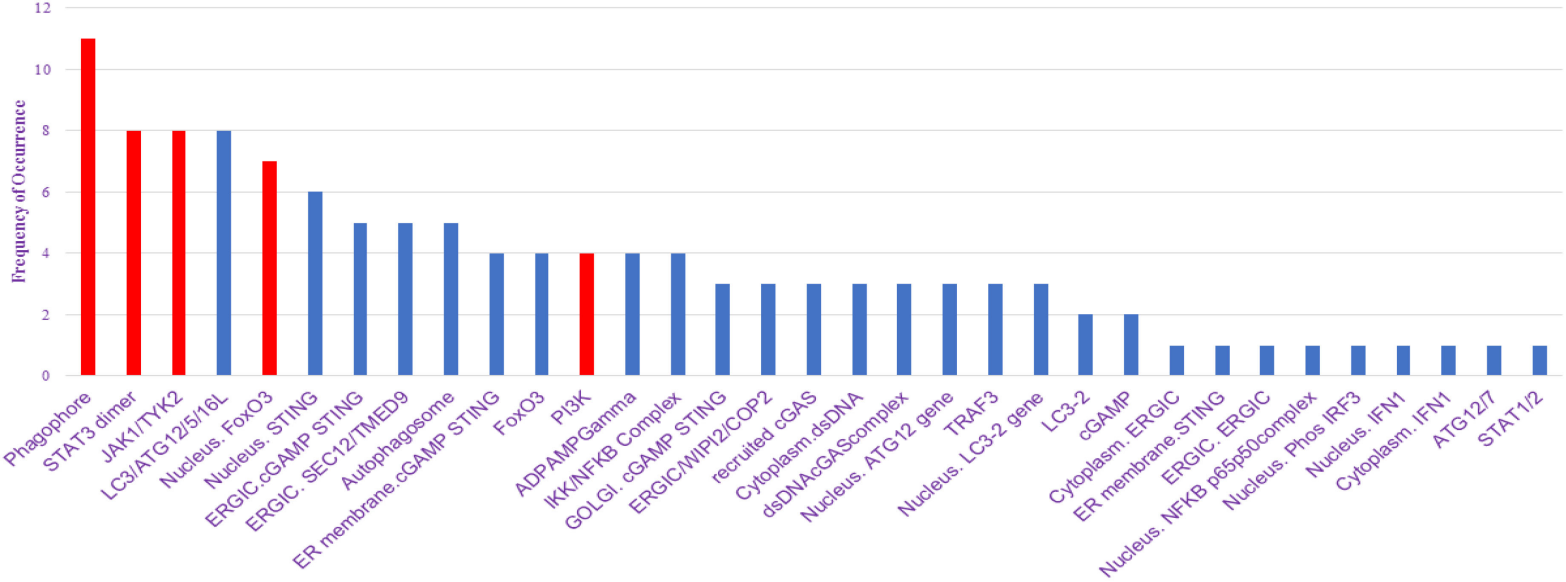
Frequency of occurrence plot to identify top nodes in the network.

We have utilized CytoHubba’s integrative approach to extract and visualize the networks present in the NSCLC mathematical model with the combination of BiNGO, another plugin of Cytoscape. The primary goal of analyzing the network properties of genes associated with NSCLC is to unravel critical information underlying NSCLC which provides a holistic view of the molecular interactions that dictate NSCLC progression. While Cytoscape helps to visualize the mathematical model in network, Cytoscape plugins such as CytoHubba and BiNGO offer analysis of the biological network via various parameters which include closeness, betweenness, bottleneck, clustering coefficient, degree, DMNC, eccentricity, EPC, MCC, MNC, radiality, and stress, leading to the identification of key proteins involved in disease severity. Based on these topological analysis methods of CytoHubba in Cytoscape plug-in, hub proteins are selected. The proteins identified from the CytoHubba analysis through all of the abovementioned parameters include phagophore, STAT3 dimer, JAK1/TYK2, LC3/ATG12/5/16L, nucleus FOXO3a, nucleus STING, ERGIC. cGAMP STING, ERGIC. SEC12/TMED9, autophagosome, ER membrane cGAMP STING, FOXO3a, PI3K, ADPAMP gamma, IKK/NFKB complex, GOLGI. cGAMP STING, ERGIC/WIPi2/COP2, recruited cGAS, cytoplasm dsDNA, dsDNAcGAS complex, nucleus ATG12 gene, TRAF3, nucleus LC3–2 gene, LC3-2, cGAMP, cytoplasm ERGIC, ER membrane STING, ERGIC.ERGIC, nucleus NFKB p65p50complex, nucleus Phos IRF3, nucleus IFN, cytoplasm IFN1, ATG12/7, and STAT1/2 which are presented in **Supplementary File S5**, and their frequency of occurrence is shown in **Figure 4**.

**Figure 5:**
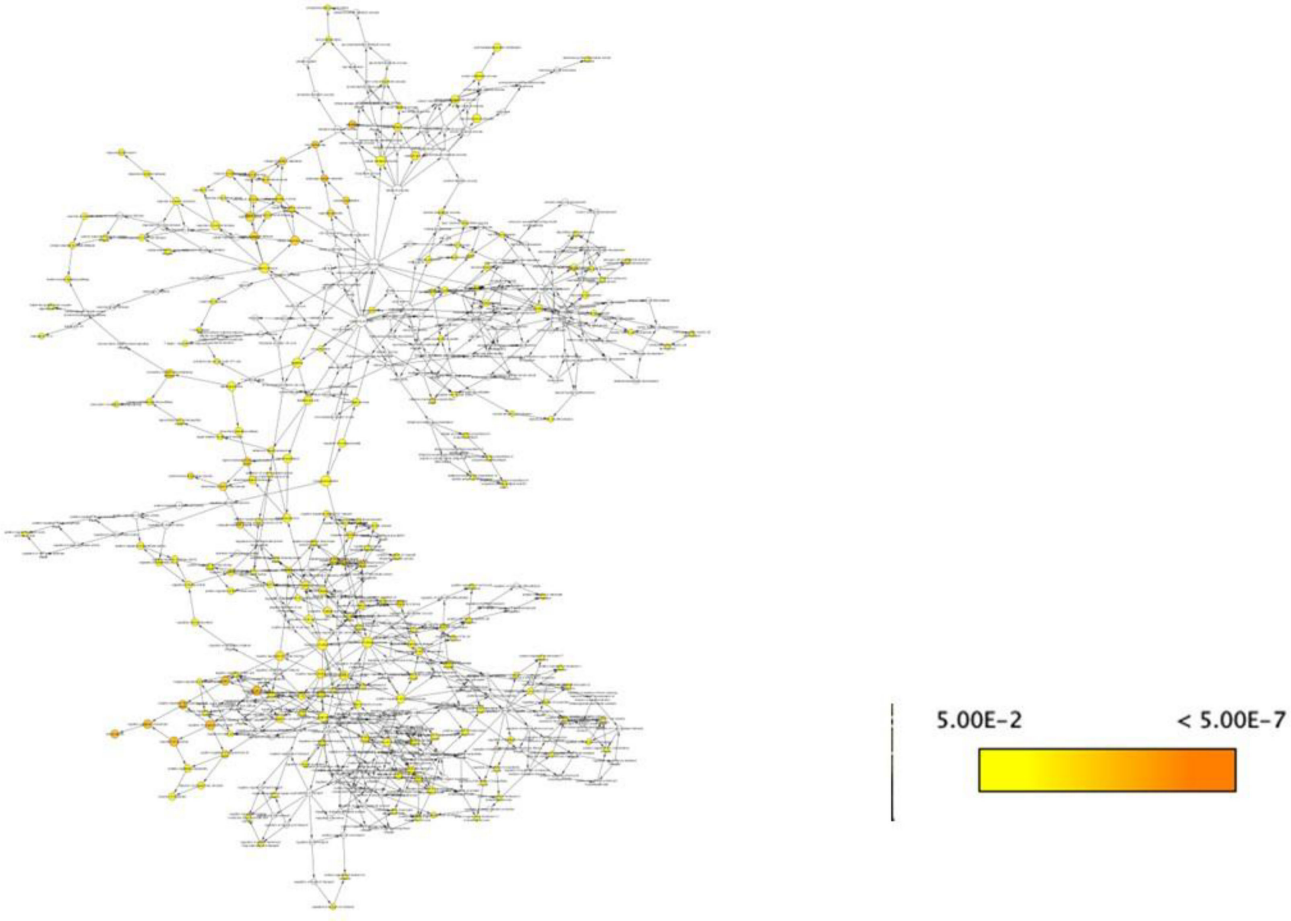
Overrepresentation analysis of the network using BiNGO.

We also analyzed our network via BiNGO, another Cytoscape plugin, to perform Gene Ontology (GO) enrichment analysis. This allowed us to identify overrepresented biological processes, molecular functions, and cellular components associated with the hub genes identified by CytoHubba. By integrating the topological network analysis from CytoHubba with the functional annotation from BiNGO, we obtained a more comprehensive understanding of the molecular mechanisms and pathways involved in NSCLC progression triggered as a result of autophagy mediated by cGAS STING and FOXO3a and how these genes are involved in cell regulation and autophagy (**Figure 5**). The list of all GO from BiNGO analysis is presented in **Supplementary File S6**.

### 3.7 Structure prediction of cGAS, STING, FOXO1, and FOXO3a

**Figure 6:**
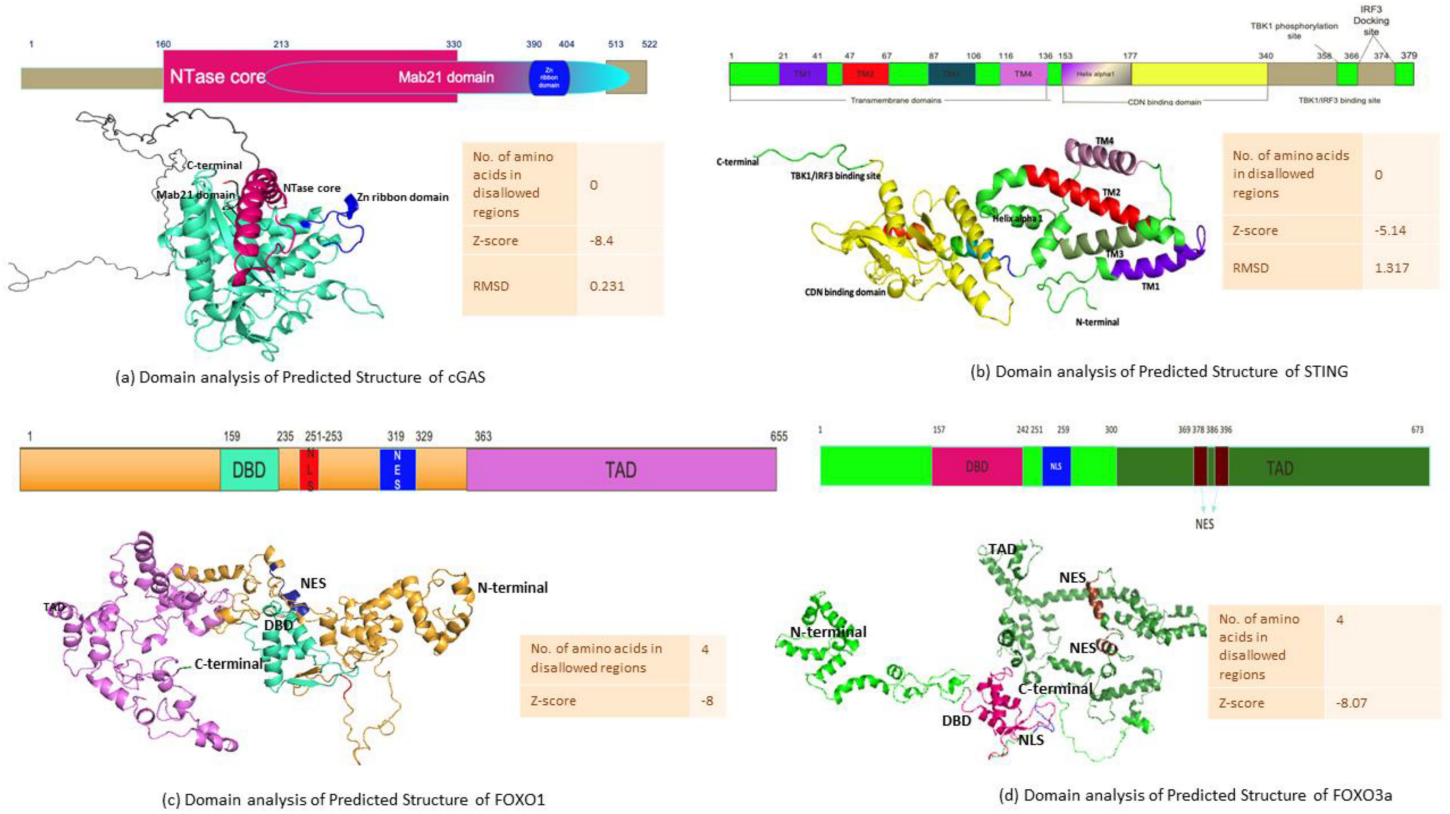
Computationally predicted structures and domain map analysis. **(a)** cGAS, **(b)** STING, **(c)** FOXO1, and **(d)** FOXO3a.

In order to understand the sequence–structure–functional properties of cGAS, STING, FOXO1, and FOXO3a, elucidating the structures of the following proteins is necessary. The structure is predicted by homology modeling for cGAS and STING where the number of amino acids in disallowed regions for both predicted structure is 0, ensuring that the structure has good stereochemical quality and lies within favorable regions of the Ramachandran plot. The predicted structure of cGAS shows a z-score of −8.4 with an RMSD of 0.231 (**Figure 6a**), while the predicted structure of STING has a z-score of −5.14 with an RMSD of 1.317 (**Figure 6b**). These z-score values fall within the range typically observed for experimentally determined protein structures of similar size, and the low RMSD values indicate a close fit between the predicted structure and reference structures, thereby supporting the reliability and accuracy of the generated structure for both cGAS and STING. The analysis of Robetta-generated structure of FOXO1 and FOXO3a revealed four proteins in the disallowed regions for both structures. The value of z-score for the predicted structure of FOXO1 is -8, while FOXO3a has a z-score value of -8.07 (**Figures 6c, d**).

### 3.8 Protein–protein interaction studies between cGAS–STING, FOXO1, and FOXO3a

The available literatures concerned with NSCLC elucidate an intricate interaction between cGAS and STING. The ClusPro 2.0 web server, a rigid docking approach, was utilized, which rotates and translates one protein (the ligand) relative to the other (the receptor). Based on energy calculations and clustering from the server, the results were filtered out to identify the most likely docked structures. The protein–protein interaction was observed between cGAS and STING protein and then between cGAS–STING complexes with FOXO1 and FOXO3a separately. The docked structure with the lowest energy has been considered for further analysis. Then, Proteins, Interfaces, Structures and Assemblies (PDBePISA), a web-based tool (available online: https://www.ebi.ac.uk/pdbe/pisa/), was used to analyze macromolecular structures, particularly focusing on interfaces between cGAS and STING.

**Figure 7:**
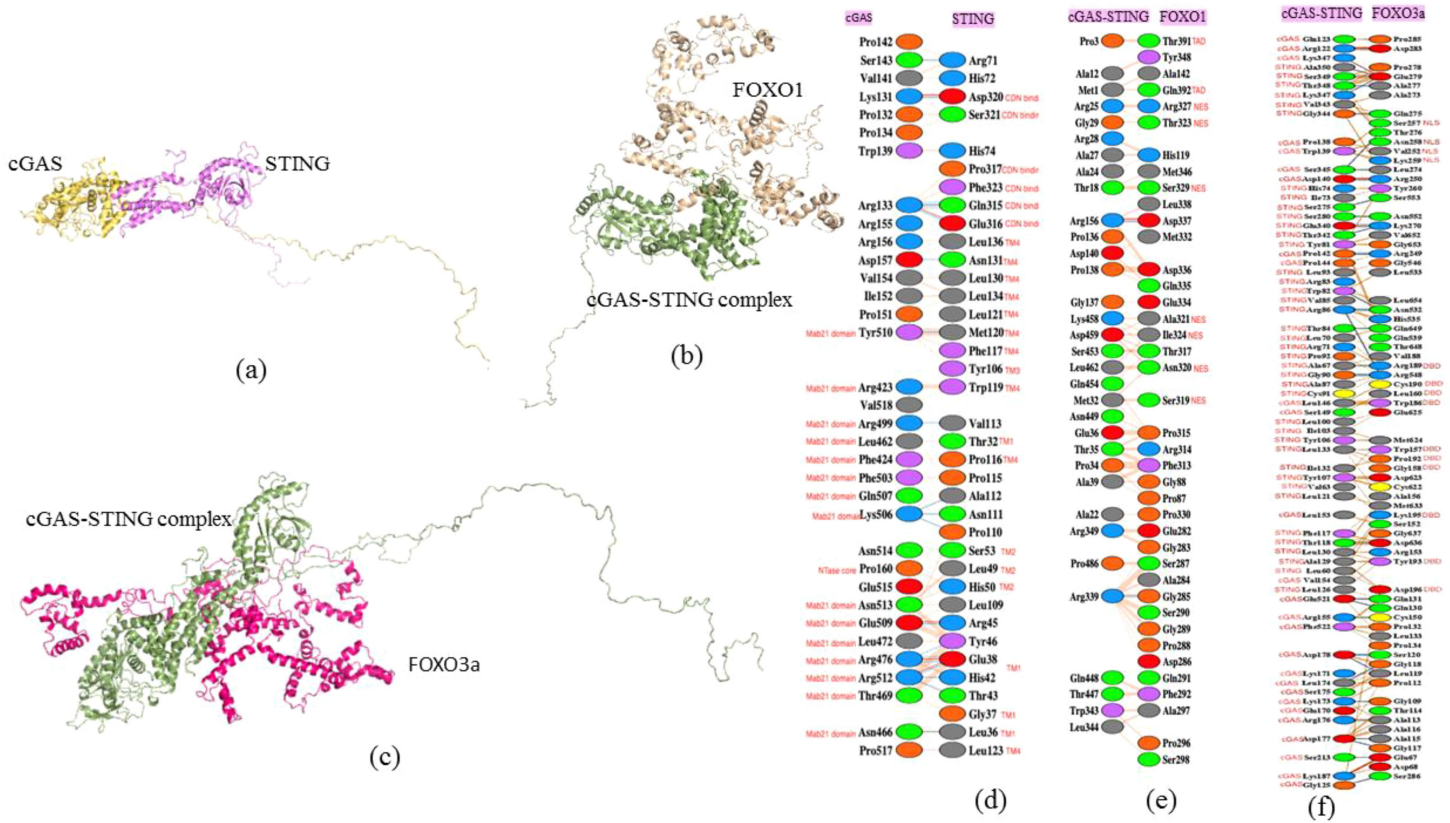
Protein–protein interaction studies using molecular docking and identifying the interacting residues. **(a)** cGAS–STING docked protein complex. **(b)** cGAS–STING–FOXO1 docked protein complex. **(c)** cGAS–STING–FOXO3a docked protein complex. **(d)** Interacting residues of cGAS and STING. **(e)** Interacting residues of cGAS–STING complex and FOXO1. **(f)** Interacting residues of cGAS–STING complex and FOXO3a.

Additionally, PDBsum enabled a detailed structural analysis of the interaction complexes, uncovering critical amino acid residues that mediate the specific binding interfaces between cGAS and STING as well as cGAS–STING complex with FOXO1 and FOXO3a (**Figures 7a–f**). This comprehensive evaluation highlighted the distinct residue-level interactions essential for the association of cGAS–STING with FOXO1 and FOXO3a, thereby emphasizing the molecular underpinnings of these protein–protein interactions. Based on the domain map of cGAS and STING, the amino acid residues of cGAS from Mab21 domain Tyr510, Arg423, Arg499, Leu462, Phe424, Phe503, Gln507, Lys506, Asn513, Glu509, Leu472, Arg476, Arg512, Thr469, and Asn466 along with Pro160 from NTase core domain interact with Asp320, Ser321, Pro317, Phe323, Gln315, and Glu316 of CDN binding domain of STING. Other interfacial residues of STING interacting with cGAS belong to TM4, including Leu136, Asn131, Leu130, Leu134, Leu121, Met120, Phe117, Trp119, Pro116, and Leu123, and then Tyr106 from TM3 domain, Ser53, Leu49, and His50 from TM2 domain, and Glu38, Gly37, and Leu36 from TM1 domain (**Figures 7a, d**).

**Figure 8:**
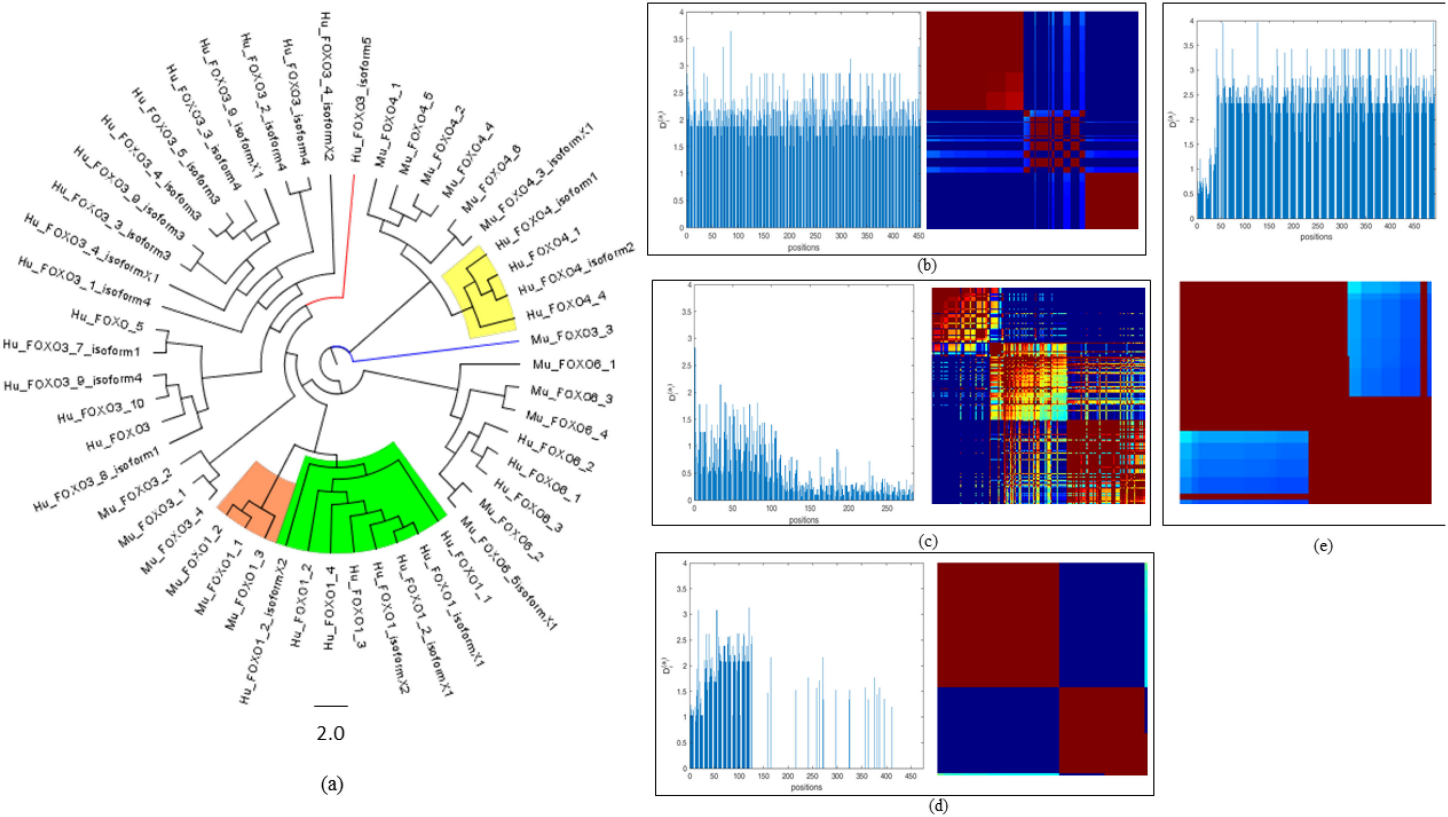
Amino acid sequence conservation studies of cGAS, STING, and FOXOs. **(a)** Phylogenetic tree of FOXO1, FOXO3a, FOXO4, and FOXO6 protein of *Homo sapiens* and *Mus musculus*. **(b)** cGAS protein in *Homo sapiens* and *Mus musculus* at 0.5 cutoff. **(c)** STING protein in *Homo sapiens* and *Mus musculus* at 0.2 cutoff. **(d)** FOXO1 protein in *Homo sapiens* and *Mus musculus* at 0.5 cutoff. **(e)** FOXO3a protein in *Homo sapiens* and *Mus musculus* at 0.5 cutoff.

Furthermore, the interfacial residues between cGAS STING complex and FOXO1 were identified. PDBsum results revealed that the interfacial residues of FOXO1 included Thr391 an Gln392 from TAD domain and Arg327, Thr323, Ser329, Ala321, Ile324, Asn320, and Ser319 from NES domain of FOXO1 (**Figures 7b, e**). The interfacial residues between cGAS–STING complex and FOXO3a were also studied to understand the downstream signaling associated with autophagy in NSCLC. The interacting residues involved- Ser257, Asn258, val252, and Lys259 from NLS domain and Arg189, Cys190, Leu160, Trp186, Trp157, Pro192, Gly158, Lys195, Tyr193, and Asp196 present in DBD domain of FOXO3a (**Figures 7c, f**).

The Gibbs free energy (ΔG) value which measures the amount of energy available in a chemical or physical process at constant temperature and pressure for cGAS–STING docking was -12.8 kcal/mol, indicating strong complex formation due to spontaneous reaction. The ΔG observed was -35.4 kcal/mol for cGAS–STING complex and FOXO1 docking. The observed ΔG for cGAS–STING complex docked with FOXO3a was -18.6kcal/mol.

### 3.9 Phylogeny analysis of FOXOs

Phylogenetic tree analysis revealed that an isoform of FOXO1 from *Homo sapiens* (Hu_FOXO1_3) emerged as a distinct outlier, indicating divergent evolutionary behavior relative to other members of the FOXO1 protein family with bootstrap value 0.5. In the case of FOXO3a protein of *Homo sapiens*, with the phylogeny analysis, we inferred that some proteins evolved from separate ancestors, and two FOXO proteins that were named as Hu_FOXO3a_isoform5 and Hu_FOXO3a_8_isoform1 have evolved separately as outliers, supported by bootstrap value 1.1. Next, we performed the sequential conservation analysis for FOXO3a and FOXO1 protein for *Homo sapiens* and *Mus musculus*. It revealed that *Homo sapiens* FOXO protein (Hu_FOXO3a_isoform5) and FOXO3a protein of *M. musculus* have common ancestors, and the clustering of human and mouse isoforms within common clades underscores the evolutionary conservation of key functional domains and supports orthology and the validity of cross-species functional studies as well. Mu_FOXO3a_3 and Hu_FOXO3a_isoform5 appear as outliers in the phylogeny tree (**Figure 8a**) (**Supplementary File S7**).

### 3.10 Statistical coupling analysis of cGAS, STING, FOXO1, and FOXO3a sequences in *Homo sapiens* and *Mus musculus*

To identify co-evolving residues and functionally coupled regions within cGAS, STING, FOXO1, and FOXO3a, SCA was performed on aligned sequences to quantify the evolutionary constraints acting on individual residues as well as their co-evolving statistical dependencies across the alignment. The analysis revealed distinct sectors—red, blue, and green—underlie the functional architecture of the protein. Since the cutoff for cGAS protein was 0.5 when *Homo sapiens* and *Mus musculus* were considered, 46 residues are present in the blue sector, 76 are in the green sector, and 78 residues which are strongly co-evolving are in the red sector (**Figure 8b**). To study the co-evolving residues for STING protein at cutoff 0.2, maximum residues that were strongly co-evolving included 78 residues in the red sector, while 46 residues are in the blue sector and 76 residues are in the green sector (**Figure 8c**). The analysis of FOXO1 proteins was performed at the 0.5 cutoff which depicts that most of the residues (68) were present in the blue regions, i.e., least conserved regions. A total of 24 residues in FOXO1 were present in the red sector, determining its statistical significance at strongly co-evolving residues, while 25 reside depicting mixed conservation and are present in the green sector (**Figure 8d**). The cutoff was set to 0.5 for the analysis of FOXO3a proteins present in *Homo sapiens* and *Mus musculus*, the analysis of which reveals that 26 residues were present in the blue sector and 13 residues were in the green sector (**Figure 8e**). Furthermore, with regard SCA for protein sequences of FOXO1, FOXO3a, FOXO4, and FOXO6 for *Homo sapiens* and *Mus musculus* at the cutoff of 0.2, the results depict that a total of 71 residues are strongly co-evolving and thus are present in the red sector, 17 residues are present in the blue sector, and 43 residues are present in the green sector.

### 3.11 Immunofluorescence and confocal microscopy

**Figure 9:**
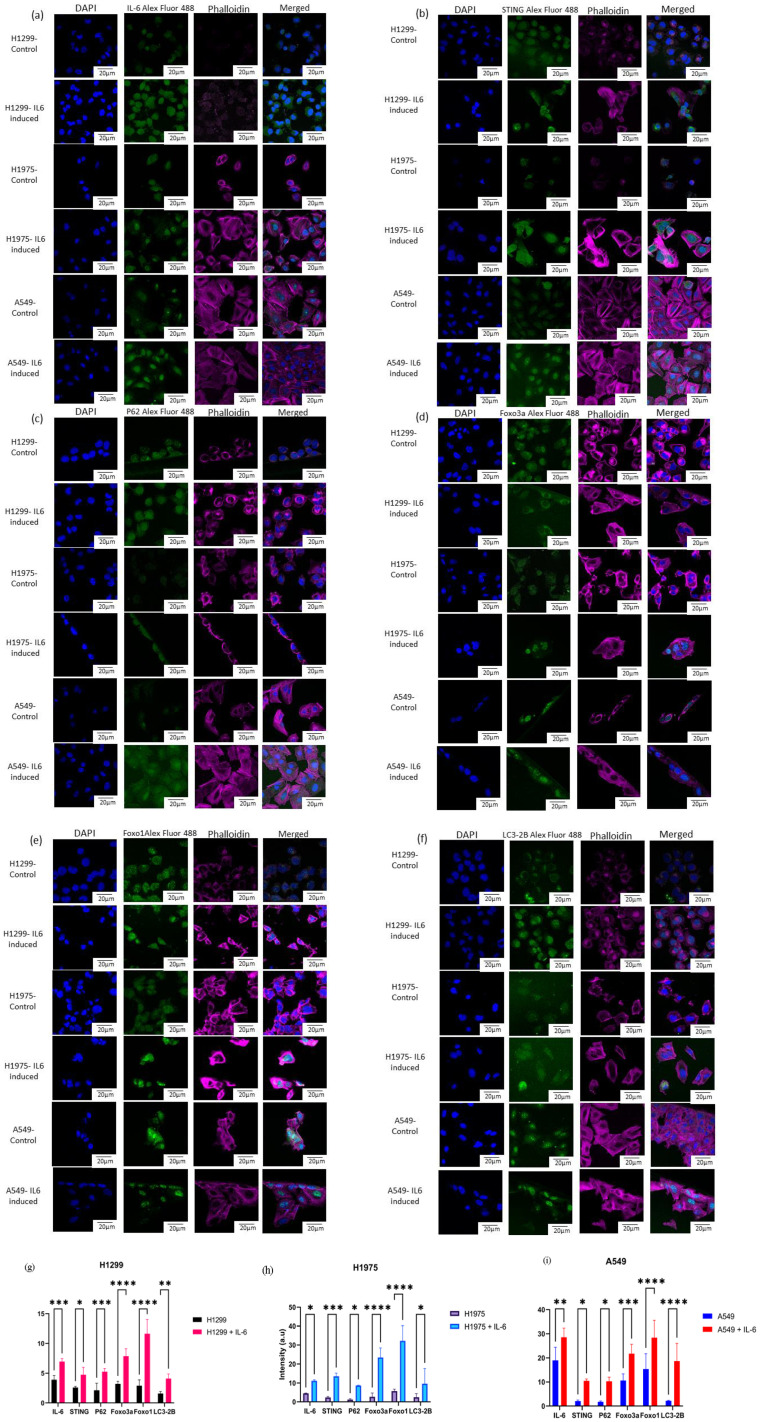
Laser scanning. confocal microscopy was performed on H1299, H1975, and A549 cell lines, both with and without IL-6 stimulation. Single-channel images captured DAPI (blue) for nuclear staining, phalloidin (magenta) for cytoskeletal visualization, and antibody staining (green), along with merged composite images of all channels. The antibodies used for immunofluorescence detection included **(a)** IL-6, **(b)** STING, **(c)** P62, **(d)** Foxo3a, **(e)** Foxo1, and **(f)** LC3-2B. Quantitative analysis of fluorescence intensity was conducted for each protein across the cell lines under control and IL-6-induced conditions. The results are graphically represented for **(g)** H1299 and IL-6-treated H1299, **(h)** H1975 and IL-6-treated H1975, and **(i)** A549 and IL-6-treated A549 cells. Statistical comparisons were performed using two-way ANOVA followed by Sidak’s multiple-comparison test, with a p-value *p < 0.05, ** p < 0.01 , ***p < 0.001 , ****p < 0.0001 considered statistically significant.

Upon treatment with IL-6, immunofluorescence studies revealed that all three lung cancer cell lines H1299, A549, and H1975 demonstrated significant alterations in the expression levels of several key markers as compared with their control counterparts. IL-6 stimulation resulted in a significant increase in the intensity of IL-6 itself (**Figure 9a**), STING (**Figure 9b**), P62 (**Figure 9c**), Foxo3a (**Figure 9d**), Foxo1 (**Figure 9e**), and LC3-2B (**Figure 9f**), with the most pronounced expression observed for Foxo3a and Foxo1 on the induction of IL6. The same trend was observed in A549 and H1975 cell lines, where IL-6 exposure robustly elevated the levels of the assayed targets. Notably, statistical analysis revealed high levels of significance (ranging from *p* < 0.05 to *p* < 0.0001) for the upregulation of most markers across all cell lines, especially for Foxo3a, Foxo1, and LC3-2B.

The data indicate that IL-6 not only promotes its own expression but also activates the STING pathway (as evidenced by increased STING expression) and enhances autophagic flux (reflected by higher P62 and LC3-2B levels) in these lung cancer cell lines. Furthermore, the marked upregulation of both Foxo3a and Foxo1 following IL-6 treatment points toward a strong involvement of FOXO transcription factors in the cellular response to inflammatory cytokine stimulation. Collectively, these findings suggest that IL-6 signaling orchestrates a coordinated increase in pro-inflammatory, autophagic, and FOXO-related pathways in NSCLC cells, potentially contributing to tumor cell adaptation and survival mechanisms for cancerous cells under inflammatory conditions. Two-way ANOVA was used to compare control versus IL-6-induced cell expression. We observed a significant expression level increase in IL-6, STING, P62, FOXO3a, FOXO1, and LC3-2B levels in IL-6-induced NSCLC cell lines compared with their basal level control cells, which is demonstrated in graphical format (**Figures 9g–i**).

## 4 Discussion

The reconstructed mathematical model presented here offers a system-level perspective of the cGAS–STING–FOXO regulatory axis in mediating autophagy within non-small cell lung cancer (NSCLC) cells. The model integrates compartmentalization where molecular interactions and pathway crosstalk occur. This helped us in the identification of regulatory nodes that govern autophagic flux. The simulation results indicate that autophagosome formation is strongly driven by the crosstalk of cGAS–STING signaling and transcriptional regulation by FOXO1 and FOXO3a, corroborating previous experimental findings that link cGAS–STING activation to autophagy induction (48, 49). In NSCLC, this process appears to be further amplified under ATP-depleted conditions, likely due to the heightened metabolic demands of proliferating tumor cells (50). PCA revealed that cGAMP–STING, nuclear FOXO1, nuclear FOXO3a, and ATG12/5 were high-impact species with strong influence over autophagic progression along with other proteins playing a significant role in NSCLC. This aligns with prior observations that FOXO transcription factors serve as pivotal regulators of autophagy-related genes (51, 52). Moreover, the PI3K/AKT/mTOR pathway emerged as a crucial upstream modulator not only influencing ULK1 activation but also indirectly reinforcing STING expression via HIF-1α. The integration of both metabolic stress and immune surveillance pathways into the autophagy process underscores the adaptability of NSCLC cells in sustaining growth under stress conditions. While our study focused on STING-induced autophagy, recent studies have revealed that STING also induces necroptosis, a caspase-independent programmed cell death, via the ZBP1–RIPK3–MLKL axis, independent of TNFR1 and FADD (53). This alternative death pathway is transcriptionally primed by STING-driven type I interferon signaling, which upregulates ZBP1 and MLKL. Thus, STING serves as a dual regulator of both autophagy and necroptosis. Moreover, the pathway for endoplasmic reticulum stress and associated necroptosis has been found to be driven by triggered levels of FOXO proteins as well (54). Within NSCLC, where both STING and FOXO protein are activated under metabolic stressful or DNA-damaging conditions, these proteins under triggered conditions may together influence whether a cell undergoes autophagy-mediated survival or necroptosis-mediated death. However, STING-based downstream signaling of necroptosis outcome is likely determined by cellular context as well as FOXO-mediated stress responses. Moreover, it has been found that STING activation, apart from acting in tumor cells, also modulates its surrounding milieu. Clinical and translational studies in NSCLC have shown that STING pathway activation of peripheral blood immune cells (PBMCs) correlates with the expression of immune-related genes, CD8^+^ T-cell infiltration as well as response to immunotherapy (55). Moving toward the flux and network topology analyses, it highlighted STAT3, JAK1/TYK2, PI3K, and FOXO proteins as key components. These nodes are likely determinants in cell fate decisions, balancing pro-survival autophagy with other responses such as apoptosis or inflammation (56). Network centrality measures from Cytoscape’s CytoHubba plugin identified hub proteins. Closeness and betweenness are the classic centrality measures; closeness centrality is defined as the connectivity of a node, determining how quickly it reaches other nodes of the network and is mathematically represented as the sum of the shortest-path distances from a node to other nodes, while betweenness centrality defines the influence a node has over other nodes in the network. Bottleneck is another network metric which identifies nodes that act as critical articulation points in shortest−path routing across communities, the removal of which may lead to a significant disruption of flow and overall connectivity of the network. Degree for any node is defined as the number of direct connections (edges) a node has established in a network. It reflects the node’s immediate activity or influence within its local neighborhood, and the tendency for a node’s neighbors to be connected to each other is defined under the clustering coefficient. Density of maximum neighborhood (DNMC) is defined as how much a node is central and influential within the network, measuring the density of a node’s neighborhood. Eccentricity is calculated by taking the reciprocal of the path length which is the longest shortest-path from a given node to all other nodes, and a lower eccentricity value means that the node is more central. Edge percolated component (EPC) is calculated as the average of whether the edges remain connected in the reduced networks based on the assigned probabilities when edges are removed randomly. Since a network comprises of multiple nodes, when these nodes are directly connected, they make a larger network; these subgraphs are known as clique and quantified in terms of maximal clique centrality (MCC) to define the nodes which are central and are likely to be hub genes. Another topological analysis for a node is done with the help of maximum neighborhood component (MNC) which identifies its largest connected component. Radiality, unlike eccentricity, considers all shortest paths and defines how a node is connected to all other nodes. In order to define key regulators in the biological network, analyzing the number of times any node appears on the shortest paths between all other possible pairs is crucial, which is defined under the stress centralities. Considering all of the parameters stated above, the biological network was analyzed, and nodes with high frequency of occurrence were highlighted. Furthermore, BiNGO-based functional annotation confirmed GO enrichment of autophagy, immune signaling, and cellular stress response.

Structure modeling and protein–protein docking analysis revealed stable interactions between cGAS and STING as well as between the cGAS–STING complex and FOXO1 and FOXO3a transcription factors. The predicted ΔG value for FOXO1 binding was particularly high, suggesting that FOXO1 may form a more stable complex with cGAS–STING than FOXO3a, potentially resulting in differential transcriptional activation profiles. This highlights the importance of studying the FOXO3a/FOXO1 axis in the context of cGAS STING in NSCLC.

Furthermore, interface residue mapping uncovered domain-specific interactions such as FOXO1 NES domain and FOXO3a DBD domain contacts that could be targeted for selective disruption. Such structural insights open avenues for a novel drug design that may play a significant role in modulating the cGAS–STING signaling-mediated autophagy axis which may be regulated by FOXO1 and FOXO3a in NSCLC. However, while these computational predictions provide valuable structural interactions, they are inherently limited by static docking. So, for a clear conceptualization, biochemical validation through co-immunoprecipitation (co-IP) or pull-down assays will be essential to confirm the physical interaction between FOXO proteins and the cGAS–STING complex and to characterize its regulatory relevance in NSCLC.

Phylogenetic analysis provided evolutionary context, revealing that although there are different isoforms of FOXO family members, there are outliers which were divergent isoforms of FOXO1 and FOXO3a in *Homo sapiens* and *Mus musculus*. The conservation of FOXO isoforms in both species thereby supported cross-species conservation sequence studies. Using SCA, we identified a high number of co-evolving clusters in cGAS and the transmembrane domain of STING that are critical for functional specificity, further refining our understanding of evolutionarily conserved autophagy regulation mechanisms. We validated the cellular expression of IL-6 and STING proteins upon IL-6 induction using confocal microscopy.

Overall, this study provides a comprehensive insight of how cGAS–STING with FOXO1 and FOXO3a signaling coordinates autophagy in NSCLC. These findings strengthen the hypothesis that targeting FOXO-driven autophagy could be a viable therapeutic strategy to enhance tumor survival in NSCLC patients.

## 5 Conclusion

The mathematical modeling of cGAS–STING and IL-6 immunological signaling offers a sophisticated systems biology framework to understand their interplay with autophagy in FOXO1/FOXO3a signaling in conjunction with cGAS–STING, both of which are essential regulators of autophagy during lung cancer progression. By merging key immune-inflammatory pathways with autophagic mechanisms, the model provides deep mechanistic insights into tumor-induced autophagy dysregulation. Moreover, systems-level network analysis identifies critical regulatory nodes, particularly activation of the FOXO1–FOXO3a axis, presenting actionable targets for precision medicine strategies in NSCLC patients.

Consistent with these predictions, our experimental findings demonstrate that IL-6 robustly upregulates the expression of FOXO3a, FOXO1, STING, and autophagy markers (P62, LC3-2B) across diverse NSCLC cell lines. The observed induction of the FOXO axis and autophagy-related proteins upon IL-6 treatment underscores the tight functional linkage between inflammatory cytokine signaling, cGAS–STING pathway activation, and FOXO-driven autophagic responses. These results further support the model’s assertion that IL-6 and cGAS–STING signaling converge on FOXO transcription factors to coordinate autophagy, immune modulation, and survival in lung cancer cells. Thus, targeting the IL-6–STING–FOXO signaling axis may offer novel, effective therapeutic approaches to manipulate autophagy and improve outcomes in NSCLC.

## Data Availability

The original contributions presented in the study are included in the article/**Supplementary Material**. Further inquiries can be directed to the corresponding author.
